# Advertising in health and medicine: using mass media to communicate with patients

**DOI:** 10.1186/s12913-020-05599-3

**Published:** 2020-09-15

**Authors:** James K. Elrod, John L. Fortenberry

**Affiliations:** 1 Willis-Knighton Health System, 2600 Greenwood Road, Shreveport, LA 71103 USA; 2 LSU Shreveport, 1 University Place, Shreveport, LA 71115 USA

**Keywords:** Advertising, Marketing communications, Promotion, Hospitals, Healthcare

## Abstract

**Background:**

Advertising—a marketing communications method involving the paid use of mass media to deliver messages to desired audiences—represents one of the most common and effective avenues for engaging current and prospective patients. Although late to proliferate in the health services industry due to tradition, the medium of communication is now firmly established and routinely deployed by health and medical organizations far and wide. Despite widespread use, healthcare providers must take opportunities, when and where possible, to stay abreast of the latest details concerning advertising and its associated applications, increasing the likelihood of successful audience engagements.

**Discussion:**

Maintaining an awareness of current developments in health services advertising can be facilitated by acquiring operational perspectives and practices from peer institutions. Most any healthcare provider can benefit from experiential views as they can shape and hone advertising approaches. Gaining such insights can be difficult, given competitive sensitivities, but occasionally healthcare institutions are compelled to share knowledge in published accounts, with this particular article following suit. Specifically, insights and experiences from Willis-Knighton Health System’s extensive and historic use of advertising are shared, bolstering the experiential accounts available in the literature and supplying operational guidance for health and medical providers.

**Conclusions:**

Advertising, if well devised and deployed, offers healthcare providers opportunities to dramatically improve their fortunes by successfully engaging current and prospective patients, hastening exchange and building vital market share. In pursuit of advertising excellence, health and medical establishments can bolster associated endeavors by tapping into the experiences of other healthcare providers, permitting insights which might potentially be incorporated into communicative pathways. This account sheds light on the advertising operations of a major health system, supplying food for thought for the advancement of advertising acumen.

## Background

Health and medical establishments provide arguably the most essential services offered in any given community. From quality-of-life enhancements to life-saving interventions, the services provided by healthcare organizations are without parallel, making these entities key community assets [1–3]. But despite the skill of physicians, the magnitude of medical technologies, the compassion of nurses, or the benefit of any other associated investment, healthcare services—even those of exceptional quality—possess very little impact potential unless they are communicated effectively to current and potential patients [4–11].

In their quests to engage audiences, healthcare institutions generally turn to the marketing communications mix, a collection of pathways for directing messages to desired groups [7, 12, 13], with advertising being one of the most commonly utilized and successfully pursued avenues for doing so [4, 7, 12]. Advertising uses mass media (e.g., television, radio, newspaper, billboard) to deliver messages to current and prospective customer populations, with these communications being paid for by the advertising party [7, 8, 13]. Although late to proliferate in the health services industry due to tradition, this medium of communication is now firmly established and routinely deployed by health and medical organizations far and wide [4, 6, 14, 15].

Advertising offers great potential for establishing effective dialogues with customers, but care must be taken to devise and deploy it effectively, something which requires a keen understanding of its characteristics and exceptional attention to detail on operational fronts. As such, healthcare providers must take opportunities, when and where possible, to stay abreast of the latest details concerning advertising and its associated applications and strategies, increasing the likelihood of successful audience engagement endeavors [8, 9, 15].

Maintaining an awareness of current developments in health services advertising can be facilitated by acquiring operational perspectives from peer institutions. Gaining such insights can be challenging, given competitive sensitivities, but occasionally healthcare establishments are compelled to share associated knowledge in published accounts, with this particular article following suit. Specifically, insights and experiences from Willis-Knighton Health System’s extensive and historic use of advertising are shared, bolstering the experiential accounts available in the literature and supplying operational guidance for health and medical providers.

## Definition and overview

To best understand advertising, it is helpful to first gain an awareness of its context and placement in the greater discipline of marketing. Formally defined, marketing is “a management process that involves the assessment of customer wants and needs, and the performance of all activities associated with the development, pricing, provision, and promotion of product solutions that satisfy those wants and needs” [7], p. 288. As presented in this definition, promotion is a prominent component of marketing, something further illustrated by its inclusion as one of the Ps in the classic expression known as the *four Ps of marketing* (i.e., Product, Price, Place, Promotion). The promotion aspect of marketing essentially entails any and all elements associated with engaging audiences, with the core pathways for engagement being depicted in a descriptive model known as the marketing communications (or promotions) mix [7, 16].

Classically illustrated, the marketing communications mix contains five principal avenues of communication; namely, advertising (i.e., the paid use of mass media to deliver messages), personal selling (i.e., the use of sales agents to personally deliver messages), sales promotion (i.e., the use of incentives, such as contests and free giveaways, to encourage patronage), public relations (i.e., the use of publicity and other unpaid promotional methods to deliver messages), and direct marketing (i.e., the delivery of messages via mail, the Internet, and similar routes directly to consumers) [7, 8]. Healthcare institutions evaluate each option and select one or more believed to be most capable of reaching target audiences, all for the purpose of enticing patronage or compelling some other form of meaningful exchange [7, 13].

Of the avenues identified in the marketing communications mix, advertising is perhaps the best known and first method to come to mind when considering promotions opportunities. This should not come as a surprise as advertising—formally defined as “a promotional method involving the paid use of mass media to deliver messages” [7], p. 219—is the most public of communicative avenues. Television commercials promoting the latest offerings of an urgent care center, magazine advertisements communicating the grand opening of a medical clinic, billboard advertisements presenting the current wait time at a hospital’s emergency department, and other open conveyances appear prominently and frequently across scores of communities, making advertising a familiar and robust influence easily recognized by most anyone [15, 16].

While advertising is now a mainstay of the health services industry, this was not always the case. Prior to the 1980s, advertising was viewed to be beneath the dignity of medical providers, with some also fearing its potential to impact referrals between and among caregivers, the time-honored method of patient acquisition. Further, the American Medical Association prohibited its members from engaging in advertising. During this era, the healthcare industry’s stance was quite unusual as other industries had been using advertising for decades, benefiting from its deployment. In the 1980s, however, resistance against health services advertising diminished, aided by the US Federal Trade Commission’s scrutiny of the American Medical Association’s ban on its members’ use of advertising, something which ultimately led to its relinquishment. Going forward, health services advertising began to flourish, and today, it thrives, having become a foundation of healthcare industry operations and an established component of the communications arsenal of most any health services organization [4, 6, 7, 12, 15].

## Institutional background and deployment history

With origins dating back nearly a century, Willis-Knighton Health System has faced and continues to face all of the challenges associated with delivering healthcare services, one of which involves communicating effectively with customer groups. Based in Shreveport, Louisiana and situated in the heart of an area known as the Ark-La-Tex where the states of Arkansas, Louisiana, and Texas converge, Willis-Knighton Health System holds market leadership in its served region where it delivers comprehensive health and wellness services through multiple hospitals, numerous general and specialty medical clinics, an all-inclusive retirement community, and more. The prized position of market leadership did not occur overnight; it instead resulted from painstaking efforts on multiple fronts, with associated growth pursuits largely beginning in the 1970s as part of a comprehensive expansion initiative.

During this era, myriad growth-fueling innovations were pursued, including adoption of the hub-and-spoke model [17, 18], establishing centers of excellence [19], expanding physical space via the practice of adaptive reuse [20, 21], and more. Of these, one most integral to building patient volume was on the marketing communications front; namely, the early embracement of advertising as a means of engaging audiences [14–16]. Willis-Knighton Health System’s use of advertising happens to be one of the most enduring in the health services industry, as the establishment adopted the medium of communication nearly a decade prior to the industry’s broad acceptance of such in the 1980s [9, 14, 15]. Its adoption primarily was compelled by Willis-Knighton Health System’s desires to more effectively and reliably communicate its offerings to the public, something that the primary communications vehicle of the day, public relations, routinely fell short of achieving. As a result of this early experimentation, Willis-Knighton Health System gained advertising proficiencies which advanced its strategic and tactical communicative prowess. This afforded an enduring competitive advantage, which even today, years after advertising’s arguably universal acceptance in the health services industry, continues to amplify the institution’s marketing communications efforts [14, 15].

## Context within marketing communications

**Fig. 1:**
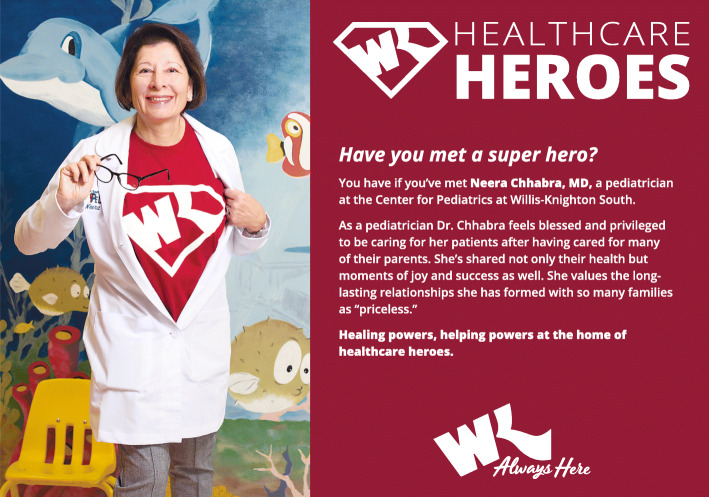
A newspaper advertisement promoting Willis-Knighton Health System’s “Healthcare Heroes” campaign

**Fig. 2:**
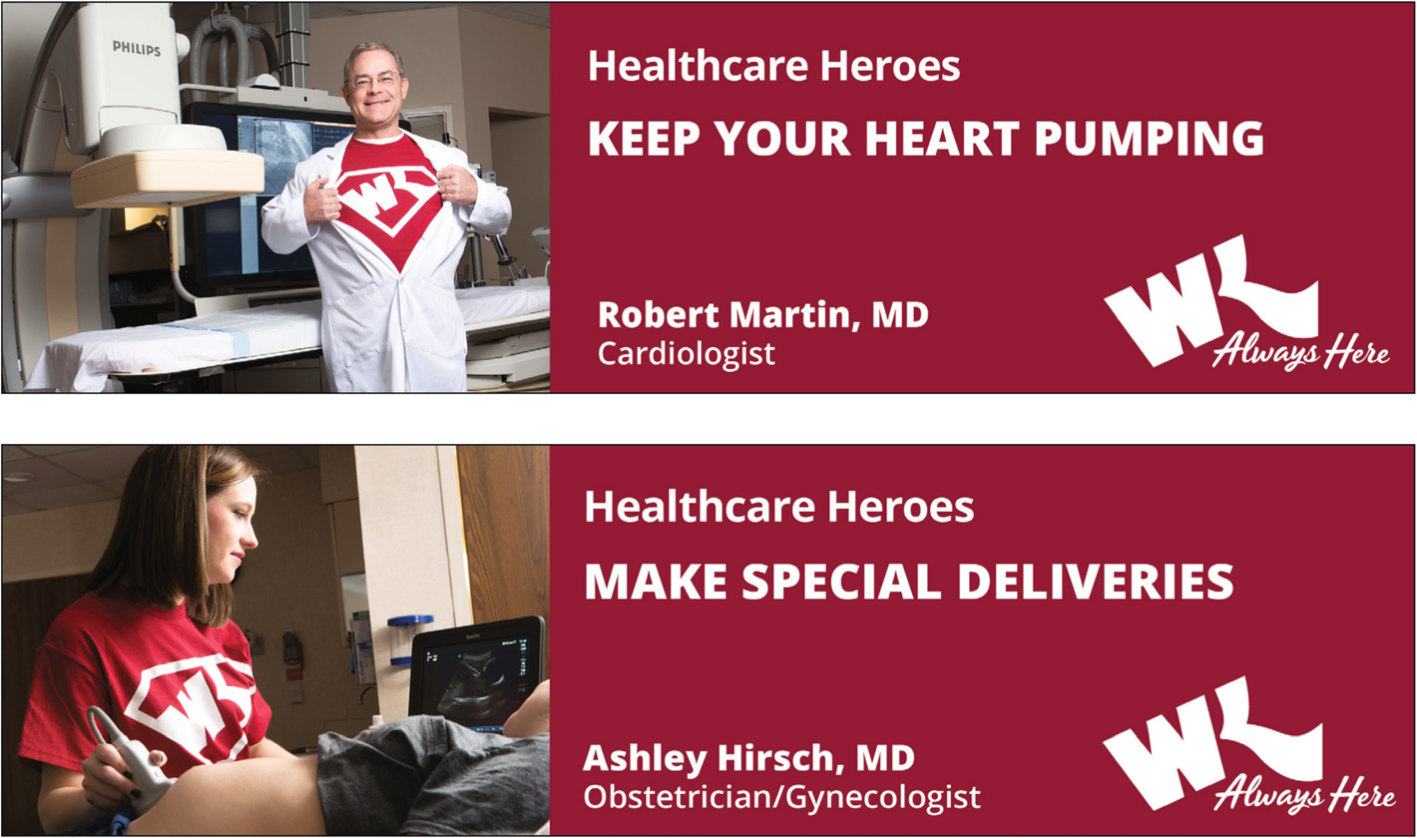
Billboard advertisements promoting Willis-Knighton Health System’s “Healthcare Heroes” campaign

In engaging current and prospective patients, Willis-Knighton Health System makes use of the full range of the marketing communications mix. Of the components of the mix, advertising constitutes the institution’s most significant communications investment [15], and generally is the first component to be considered whenever promotions needs arise. Several examples of recent newspaper and billboard advertisements, these promoting Willis-Knighton Health System’s “Healthcare Heroes” campaign, are presented in Figs. 1 and 2, and related television commercials are available at the following link: https://www.wkhs.com/video/commercials.

Core advertising opportunities classically have been placed into three categories: print, electronic, and outdoor. Notable categories within print include newspaper and magazine advertising; within electronic, they include television, radio, and Internet advertising; and within outdoor, they include billboard, street furniture, and transit advertising. Although the particular communication routes differ in form and delivery, they each share the common bond of using mass media, permitting large (i.e., mass) audiences to be exposed to messages carried. This, of course, is a key requirement of advertising, with paid use of mass media being its most distinguishing feature, differentiating it from other components of the marketing communications mix [7, 15, 22–24].

For advertising’s part of the marketing communications mix, Willis-Knighton Health System relies on a particular combination of media. Recently, the institution has been concentrating the bulk of its advertising in print media (41% of advertising expenditures), with the majority of expenditures being directed toward newspaper advertising, followed by magazine advertising. Electronic media options consume 31% of advertising expenditures, with television being the predominant media expenditure in the category, followed by Internet advertising. Outdoor media, almost exclusively billboard advertising, consumes 28% of advertising expenditures, rounding out the allotment. The noted array of advertising media tapped for promoting the institution and its services was devised based on a combination of education and experience, yielding keen insights on media effectiveness.

Of course, the formulation of Willis-Knighton Health System’s media mix is not static. Instead, it changes as media, markets, and people change in an effort to ensure that communications are properly tailored to current conditions. While alterations of the institution’s media mix historically have been subtle, environmental circumstances occasionally require more comprehensive modifications. One such environmental change, occurring gradually in recent years and continuing to evolve, involves the proliferation of computer tablets and smartphones in society. This change has dramatically altered historic patterns of information consumption, bolstering demand for electronic content, typically at the expense of print content [25–28]. This prompted Willis-Knighton Health System to begin increasing its investment in electronic advertising, most notably, Internet placements, while diminishing print advertising expenditures in concert with media trends noted in the marketplace.

Despite the clear trend toward electronic communications, the institution has proceeded cautiously in adjusting its media mix. As a comprehensive health services provider, Willis-Knighton Health System’s services appeal to virtually every segment of the population, with some groups having greater tendencies for innovation adoption than others. As such, media mix alterations have been gradual to ensure that those customer groups who are less receptive to electronic content are not neglected even as increasing numbers of people shift away from once dominant print media preferences. This particular scenario illustrates the need for healthcare providers to closely monitor environmental trends in the context of their customer populations, ensuring that findings are taken into account when formulating advertising budgets and making associated selections from among media options.

## Strengths

After decades of use, Willis-Knighton Health System has found advertising to be a highly capable avenue of communication, prompting its continued deployment as part of the institution’s overall strategy for audience engagement. Deployment justifications can be numerous, with these being dependent on the needs and desires of given healthcare organizations, but in Willis-Knighton Health System’s experience, three particular characteristics serve as primary motivations for the establishment’s use of advertising. These characteristics are as follows.

### High performance

Advertising is an age-old communicative method, arguably dating back in primitive form to the earliest days of trade. Evolving continually, its modern day proliferation is undeniable, evidenced by simple exposure to and observation of most any commercial environment. Thanks to many decades of scholarly attention, the medium of communication has been studied extensively, with findings routinely confirming its ability to effectively engage audiences. Further, its extensive and continued use by business and industry provides additional validation of the effectiveness of advertising [9, 14, 22, 23, 29]. Willis-Knighton Health System’s own experiences, some of which have been published in the scholarly literature [9, 15, 16, 30], support accounts attesting to the prowess of advertising to generate awareness and convey information effectively to current and prospective customers, giving advertising a firm place in the institution’s communications arsenal.

### Significant control

Advertising, as a paid medium of communication, is largely under the control of the advertising entity, permitting the advertiser to determine creative treatment, timing and frequency of promotion, and other attributes of given advertisements. This stands in contrast to public relations efforts which rely on press releases being accepted for presentation by media outlets, something which offers no guarantees of circulation and, even if carried, intended stories may not be communicated as desired. Ultimately, advertising, courtesy of paid placements, provides assurances that messages will be delivered as intended to desired audiences on desired timetables [15, 22, 23, 29]. As noted earlier, this particular attribute served as the primary impetus for Willis-Knighton Health System’s early embracement of advertising [14, 15].

### Exceptional variety

Advertising is available in a variety of formats—print, electronic, and outdoor, with multiple communicative avenues within these broad categories—allowing advertising entities to select routes deemed most capable of reaching sought audiences. This extensive variety also permits a wide range of creative treatments and information conveyance options, affording flexibility in message assembly and presentation, yielding practically infinite opportunities for developing advertisements specifically tailored to attract and impact desired groups [7, 15, 22–24, 29].

## Limitations

Despite its many attributes, contexts of use, applications, and the like, advertising carries a range of limitations which must be factored into any decision regarding its deployment. These limitations are as follows.

### High cost

As advertising entails the paid use of mass media, obviously, costs are involved with its deployment. Such expenses can be significant, with costs generally rising as audience exposures increase. Generally, advertisements placed in larger markets are pricier than those placed in smaller markets, given audience size differences [8, 22, 23, 29]. Even in particular markets, pricing variation typically exists when circulation differs between placements. A billboard advertisement situated on a roadway with a low traffic count, for example, will usually cost less than an equivalently-sized one located on a heavily-traveled route in a given city. But beyond the quantity of individuals exposed to a given advertisement (i.e., reach), advertising impact is also influenced by the number of times individuals are exposed to the associated advertisement (i.e., frequency), with this also acting to increase advertising expenditures [7, 8]. Despite associated costs, advertising should be considered to be an investment and, with proper planning and implementation to ensure that prudent advertising decisions are made, generating a desired return on investment is entirely possible.

### Engagement constraints

Advertising generally is considered to be one-way communication; the advertiser sends a message to audiences via a given channel of communication, but the audience cannot respond directly via that particular channel [7, 8, 12, 15, 22, 23, 29]. A television commercial or billboard advertisement, for example, can convey a promotional message, but message recipients cannot, in turn, use the given communications pathway to ask questions, indicate interest, or forward patronage decisions. (It should be noted that advertising is sometimes confused with direct marketing of which some forms do permit two-way communication, such as telemarketing, but these pathways do not meet the technical definition of advertising.) The unidirectional communication weakness of advertising, however, can be resolved by including response avenues, such as toll-free telephone numbers, website addresses, and other response mechanisms in given promotional messages. This, however, must remain at the forefront of the minds of those charged with assembling advertisements in order to maximize engagement opportunities.

### Potential for intrusiveness

If well planned and implemented, health services advertising can provide invaluable assistance to target audiences, advancing their awareness of medical offerings, educating them on healthy practices, and encouraging them in other positive manners [7, 8, 14–16]. But occasionally healthcare establishments make poor advertising decisions, selecting highly disruptive avenues which have the potential to generate animosity among message recipients. Willis-Knighton Health System is particularly vigilant in ensuring that media selected, messages conveyed, and contexts delivered are as unobtrusive as possible so as to avoid generating ill will among audiences.

The institution’s extensive use of billboard advertisements illustrates this quite well. Billboards are highly effective, yet generally free of disruptive influences. Passersby can choose to consume the information or simply look away. Contrast this with some Internet advertisements which overtake web browsers, forcing viewers to wait until the message concludes or take action (e.g., clicking a button to close advertisements) to get back to their intended pursuit, generating negative feelings which can be attributed not only to the type of advertisement, but also the establishments featured in them. Willis-Knighton Health System has a long-standing tradition of making media selections which are respectful of audiences, factoring their tastes and preferences into each and every promotions decision, something advised for any healthcare establishment engaged in advertising.

## Operational reflections

Operationally, advertising requires foundational assets similar to those required by most any intensive organizational undertaking. These resources include (1) top leadership support and commitment, (2) financial resources sufficient for funding endeavors, (3) competent personnel charged with effecting given initiatives, and (4) formal processes permitting effective planning, implementation, and evaluation of initiatives. As the viability of advertising endeavors largely depends on the availability and adequacy of foundational assets, healthcare establishments must take steps to secure them prior to engaging in advertising pursuits. Communicative needs can arise at any time, so health and medical providers, even those not currently engaged in advertising, should ensure that capable resource frameworks are developed and available on demand. With these resources in place, the stage is set to effect advertising proficiently, generating desired communications utility that fosters interest and attention on the part of target audiences, leading to all-important exchange, and potentially, long-term loyalty. Willis-Knighton Health System’s leadership, well known for pursuing innovations [31], initiated the establishment’s foray into advertising, with this ensuring the availability of foundational resources. Indeed, if top leadership support and commitment can be acquired, procurement of the remaining resources required for advertising success becomes entirely possible.

Beyond securing foundational resources and following the advisories presented elsewhere in this article, operationally Willis-Knighton Health System recommends the use of triangulation to ascertain advertising value. Advertising, of course, is a means to an end, with its goal being to generate some form of desired action on the part of target audiences. That said, the ultimate question emerging from a healthcare organization’s associated pursuits is whether its given advertisements actually delivered value (i.e., “Did our ads work?”). This might appear to be a problem-free inquiry, but determining advertising return on investment with any degree of certainty actually is quite difficult [32, 33]. This is due to the fact that many externalities exist which influence patronage decisions.

Assume that an urgent care center decided to initiate a 1-month advertising campaign and, by the month’s conclusion, observed a 10% increase in patient volume. The advertising campaign might have been the source of the increase, but other environmental circumstances, too, could have played a role. Suppose the urgent care center’s top competitor happened to be located on a roadway that was being widened at the time, creating access difficulties for patients. Suppose a sporting venue near the center was hosting a major event, drawing populations from outside of the market, some of whom might have had urgent care needs and patronized the center simply due to its convenience. Suppose a highly-regarded physician had been hired by the center at the advertising campaign’s initiation and immediately began drawing his clientele, bolstering volume, accordingly. Such events and occurrences obviously hardship advertising performance assessments. Even direct inquiries forwarded to patients querying them on advertising impact can be unreliable due to mistakes, memory lapses, and other distortions leading to inaccuracies. Difficulties in ascertaining advertising effectiveness prompted Willis-Knighton Health System very early in its associated endeavors to adopt an approach best described as triangulation, whereby a number of metrics are studied, giving indications of advertising performance.

In the case of the urgent care center promoting its services, in order to assess advertising impact, the establishment might, for example, monitor telephone, email, and in-person inquiries, assessing volume and asking parties how they heard about the center. The same could be done for actual patient encounters. Further, mechanisms could be planted within advertisements which could help determine campaign effectiveness. For example, a particular telephone number could be featured in advertisements—one unique to the given campaign and unused for other purposes—giving reasonable assurances that those inquiring via that telephone number had been exposed to the advertising message. Similarly, audiences could be prompted by given advertisements to take a particular action revealing their exposure (e.g., “Be sure to mention this ad when you make your appointment!” or “Mention this ad when you visit to receive a free gift!”). Further insights could be achieved by conducting a community survey, assessing degree of awareness of the center, providing yet another indication of the power of the noted advertising campaign. Importantly, the center’s personnel, especially its leadership, must carefully monitor the environment in an effort to detect externalities which might impact metrics, warranting their consideration when evaluating advertising performance. These and similar mechanisms can shed significant light on advertising effectiveness, helping to ascertain value, justify expenditures, and bolster knowledge which can help improve future advertising campaigns.

Triangulation indeed affords Willis-Knighton Health System with valuable intelligence, permitting access to a range of indicators that aid in determining the return on investment generated by its advertising. This approach also carries another benefit that should not be overlooked. Beyond providing indicators of effectiveness at the conclusion of campaigns, triangulation, courtesy of active monitoring, can be used to assess advertising effectiveness in real-time, as campaigns progress. This is especially helpful for lengthy campaigns running over many months. If active advertising campaigns are found to be successfully delivering value, they can be continued. If not, the campaigns can be modified in an effort to elevate performance to desired levels. Active monitoring, coupled with expedient alterations, when and where warranted, can effectively diminish the prospects of realizing failed campaigns. Mastery of triangulation goes hand-in-hand with mastery of advertising.

## Conclusions

Advertising, if well devised and deployed, offers healthcare providers opportunities to dramatically improve their fortunes by successfully engaging current and prospective patients, hastening exchange and building vital market share. As such, health and medical establishments must strive to develop proficiencies in using advertising, something especially important, given the competitive intensity which characterizes the health services industry. Beyond intensive study of advertising and experience gained through its deployment, healthcare providers can bolster their advertising skills and abilities by tapping into the experiences of other healthcare providers, permitting insights which might potentially be incorporated into communicative pathways. This particular account shed light on the advertising operations of Willis-Knighton Health System, supplying food for thought for the advancement of advertising acumen.

## Data Availability

Not applicable.
